# Biosynthesis and Genetic Regulation of Proanthocyanidins in Plants

**DOI:** 10.3390/molecules13102674

**Published:** 2008-10-28

**Authors:** Fei He, Qiu-Hong Pan, Ying Shi, Chang-Qing Duan

**Affiliations:** Center for Viticulture and Enology, College of Food Science & Nutritional Engineering, China Agricultural University, Beijing, 100083, P.R. China; E-mails: wheyfey@yahoo.com.cn (F. H.), panqiuhong2007@vip.sohu.com (Q-H. P.), shiy@cau.edu.cn (Y. S.)

**Keywords:** Proanthocyanidins, Condensed Tannins, Structures, Flavonoid Biosynthesis, Transport Factors, Regulatory Genes

## Abstract

Proanthocyanidins (PAs), also known as condensed tannins, are a group of polyphenolic secondary metabolites synthesized in plants as oligomers or polymers of flavan-3-ol units via the flavonoid pathway. Due to their structural complexity and varied composition, only in the recent years has the study on the biosynthesis and regulation of PAs in plants taken off, although some details of the synthetic mechanism remain unclear. This paper aims to summarize the status of research on the structures of PAs in plants, the genes encoding key enzymes of biosynthetic pathway, the transport factors, the transcriptional regulation of PA biosynthesis and the genetic manipulation of PAs. The problems of this field were also discussed, including the nature of the final “enzyme” which catalyzes the polymerization reaction of PAs and the possible mechanism of how the elementary units of flavanols are assembled *in vivo*.

## Introduction

Proanthocyanidins (PAs), also known as condensed tannins, are oligomers or polymers of flavan-3-ol units, which have been used as medicines or tanning agents by humans since ancient times and whose chemistry and synthesis have been studied for many decades [1,2]. As one of the most ubiquitous groups of all plant phenolics, PAs are synthesized via the phenylpropanoid and the flavonoid pathways, and are widespread throughout the plant kingdom, presenting diverse biological and biochemical activities, including protection against predation (herbivorous animals) and pathogen attack (both bacteria and fungal), as well as restricting the growth of neighboring plants [3,4,5,6]. PAs are also widely distributed in foods of plant origin, particularly in fruits, legume seeds, cereal grains and different beverages such as juice, wine, cider, tea and cocoa, where they contribute to the bitter flavor and astringency and have a significant influence on the mouth feel [7,8]. In recent years, considerable attention has been drawn to PAs and their monomers because of their potential beneficial effects on human health such as immunomodulatory and anticancer activities, antioxidant and radical scavenging functions, anti-inflammatory activities, cardio-protective properties, vasodilating and antithrombotic effects, UV-protective functions, etc [9,10,11,12,13,14,15]. Although PAs, extracted from various plants, especially grapes, have been widely used as nutritional supplements, their safety and potential long term toxicity still need to be further investigated to allow a systematic evaluation [16].

Nowadays, relying upon modern analytical techniques such as high pressure liquid chromatography (HPLC), mass spectrometry (MS), circular dichroism (CD) and nuclear magnetic resonance (NMR), researchers have been able to detect the low molecular weight compositions of PAs and their derivatives in either natural products or in artificial solutions, and provide a comprehensive understanding of their structures and chemical properties [17,18,19]. On the other hand, with the application of acid-catalyzed PA cleavage in the presence of a nucleophile, such as phloroglucinol, benzyl mercaptan or other nucleophilic agent, researchers can use a post-HPLC analysis to estimate PA polymers from natural products [20,21]. Furthermore, researchers have identified a series of landmarks in PA biosynthesis by means of biochemical, genetic and biogenetic methods, including the recent isolation of the gene for leucoanthocyanidin reductase (LAR), the functional identification of *BANYULS* (*BAN*, the gene for anthocyanidin reductase in *Arabidopsis thaliana*) and the discovery of a host of new biochemical and regulatory elements and transport factors [22]. However, some important questions in the field of PA biosynthesis remain unanswered. For example, how do flavan-3-ol units polymerize to produce PAs and whether this polymerization is enzyme-mediated or not, as well as what is the exact “enzyme” [1,23].

As early as the 1980s, Stafford commented on the similarities between PAs and lignins, primarily in their common origins as polyphenolic polymers and their potential functions in plant defense [24]. Dixon *et al*. highlighted this comment and further speculated on the potential commonalities between them with respect to their mode of assembly and the importance of their stereochemistry [1]. Besides these, other previous reviews summarized the foregoing achievement in almost every aspect of the study of PAs mainly in the past 20 years [2,7,9,22,23,25,26,27,28,29,30,31]. This paper will review the important concepts and updates in the research of PAs with regard to the elucidation of structure and stereochemistry, the general biosynthetic pathway, the potential polymerization mechanisms, their transport, regulation and genetic manipulation. The future trends in this field are also discussed.

## Structures of proanthocyanidins

PAs are oligomers and polymers composed of elementary flavan-3-ol units. As they occur widely in the plant kingdom and are considered the second most abundant group of natural phenolics after lignins, the structure complexity of PAs has been studied for many decades [7,32]. However, their structures were still ill defined until the recent application of modern technologies such as NMR, CD and MS. Generally, the structure variability of PAs depends upon the nature (the stereochemistry at the chiral centers and the hydroxylation pattern) of the flavan-3-ol extension and end units, the location and stereochemistry of the interflavan linkage (IFL) between the monomeric units and the degree of polymerization (DP) [1]. Additionally, derivatizations such as *O-*methylation, *O-*acylation, *C-* and *O-*glycosylation are involved in the rearrangement products of PAs [2].

**Figure 1:**
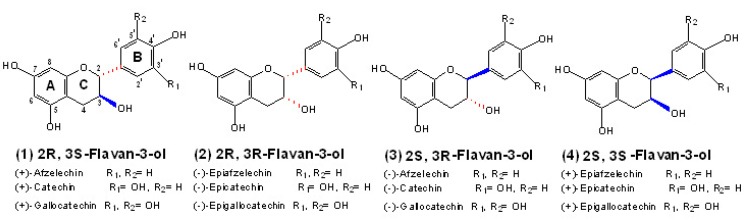
Structures of the 2*R*-type flavan-3-ols **1**, **2** and the 2*S*-type *ent*-flavan-3-ols **3**, **4**.

As the building blocks of PAs, the flavan-3-ol units have the typical C6-C3-C6 flavonoid skeletons. The heterocyclic benzopyran ring is referred to as the C ring, the fused aromatic ring as the A ring, and the phenyl constituent as the B ring [33]. They differ structurally according to the nature of the stereochemistry of the asymmetric carbons on the C rings and the number of hydroxyl groups on the B rings. 2,3-*trans*-(+)-Catechin and 2,3-*cis*-(-)-epicatechin are stated to be the most usual monomeric units in PAs, which have the opposite stereochemistry of the chiral C3 carbon on the C rings (Figure 1, **1**-**2**). As observed in (+)-catechin (2R, 3S) and (-)-epicatechin (2R, 3R), the C2configuration is almost always *R*, but some exceptions are found in monocotyledons and in selected dicotyledonous families in succession, such as *Rhus*, *Uncaria*, *Polygonum*, *Rapheolepsis* and *Schinopsis* [34,35,36], where flavan-3-ols with 2*S* type configuration are consequently distinguished by the prefix enantio (*ent-*), which may further enhance the structure complexity of PAs (Figure 1, **3**-**4**).

Acting in the early steps of the flavonoid pathway, the cytochrome P450 monooxygenases flavonoid 3’-hydroxylase (F3’H) and flavonoid 3’,5’-hydroxylase (F3’5’H) catalyze the formation of the 3’-hydroxyl and 3’,5’-hydroxyl groups on the B rings, respectively [37,38]. Thus, the presence or absence of these two enzymes determines the B-ring hydroxylation pattern of the monomeric units in PAs (Figure 1). Generally, the term proanthocyanidins refers to the release of anthocyanidins from extension positions after being boiled with strong mineral acid. Correspondingly, procyanidins designate oligomers and polymers with 3’,4’-dihydroxyl pattern ((+)-catechin and/or (-)-epicatechin units) extension units, while propelargonidins or prodelphinidins designate oligomers and polymers with extension units of 4’-hydroxyl pattern ((+)-afzelechin and/or (-)-epiafzelechin units) or 3’,4’,5’-trihydroxyl pattern ((+)-gallocatechin and/or (-)-epigallocatechin), respectively. Therefore, PAs can thus be classified, according to the differences in hydroxylation patterns, into several subgroups: propelargonidins (3,4’,5,7-hydroxyl), procyanidins (3,3’,4’,5,7-hydroxyl), prodelphinidins (3,3’,4’,5,5’,7-hydroxyl), proguibourtinidins (3,4’,7-hydroxyl), profisetinidins (3,3’,4’,7-hydroxyl), prorobinetinidins (3,3’,4’,5’,7-hydroxyl), proteracacidins (4’,7,8-hydroxyl), promelacacidins (3’,4’,7,8-hydroxyl), proapigeninidins (4’,5,7-hydroxyl) and proluteolinidins (3’,4’,5,7-hydroxyl), some of which have been synthesized by chemical methods and have not been found to date in Nature [2,30,31]. In fact, when several types of units are present in a plant species or organ they are found together in mixed polymers.

**Figure 2:**
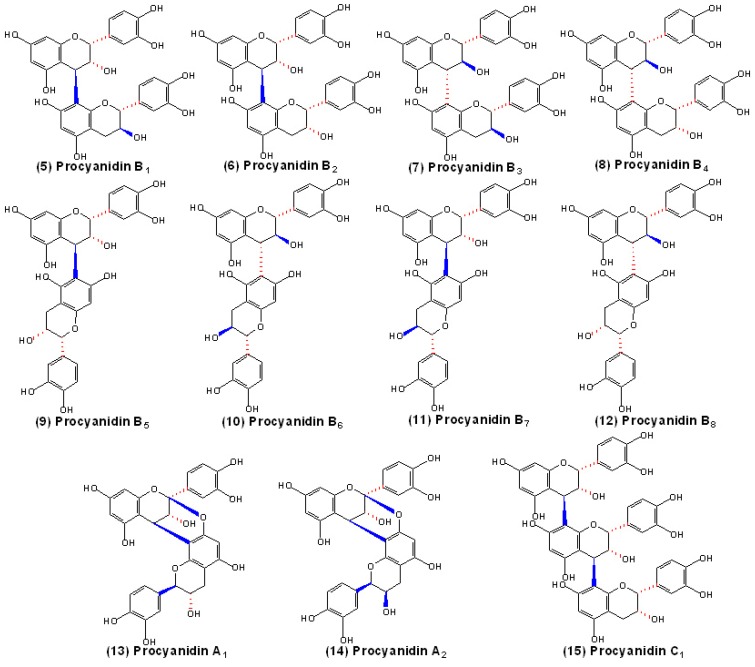
Structures of the B-type procyanidins **5**-**12**, **15** and A-type procyanidins **13**-**14**.

PAs are linked between the C4 position of the upper unit and the C8 or C6 position of the lower unit, and the type of IFL can be either α or β type. Generally, the IFL between C4 and C8 position are stereochemically predominant in procyanidins and prodelphinidins, as the C4→C8 IFL and the C4→C6 IFL are usually present in a ratio of 3:1 [2], but in 5-deoxy PAs, the C4→C6 IFL is predominant [39,40]. Oligomeric and polymeric PAs which are composed of flavan-3-ol units linked mainly through C4→C8 and/or C4→C6 IFL are categorized as B-type PAs. Among the dimers, procyanidins B1, B2, B3 and B4 are linked by the C4→C8 IFL and are the most frequently occurring in plants, whereas procyanidins B5, B6, B7 and B8 are linked by the C4→C6 IFL and are also widespread [23]. On the other hand, the flavan-3-ol units can also be doubly linked by an additional ether bond between C2 position of the upper unit and the oxygen at C7 or C5 position of the lower unit. The oligomers or polymers which contain both C2β→O→7 ether-type IFL and C4→C8 or C4→C6 IFL, as well as those which contain both C2β→O→5 ether-type IFL and C4→C6 IFL are categorized as A-type PAs, which are found in various food of plant origin, such as cranberry, plum, avocado, peanut, etc [32]. The structures of the B-type procyanidins (B1-B8, and C1) and A-type procyanidins (A1-A2) are shown in Figure 2. 

However, with more and more new PA oligomers being identified and characterized, the old nomenclature system of PAs with simple letters and numbers no longer meets the needs of researchers. As a result, it is difficult to use it to present a clear understanding of a PA’s structure [41]. A new nomenclature, derived from polysaccharides, was later introduced to name the increasing number of new structures. In this nomenclature, the elementary units of the oligomers are designated with the name of the corresponding flavan-3-ol monomers [42]. The IFL and its location and direction are indicated in parentheses with an arrow (→) and its configuration is described as α or β. In A-type PAs, both linkages are indicated within the parentheses, but it is unnecessary to indicate the oxygen in the additional ether bond since it is obvious from the substitution pattern of flavan-3-ol extension units. For instance, according to this nomenclature, procyanidin dimer B1 is named as epicatechin-(4β→8)-catechin, dimer A1 is named as epicatechin-(4β→8, 2β→7)-catechin and trimer C1 is named as [epicatechin-(4β→8)]_2_-epicatechin [23].

The degree of polymerization (DP) is another variable factor for the structure complexity of PAs. Since PAs were first elucidated in 1960s, more than 200 oligomers with DPs of no more than 5 have been well identified and characterized [7,43]. However, plant PA polymers usually have higher DPs. For example, the DP of PAs in cider apple skin and pulp range from 7 to 190, in brown or black soybean coat it can be up to 30 and even more [44,45]. Researchers also usually use mean degree of polymerization (mDP) to evaluate the molecular weight of PAs. For example, the mDP of PAs in pear is from 13 to 44, and in grape seeds it is from 2.3 to 16.7 [46,47]. Interestingly, if all constitutive units and linkages can be distributed at random within a polymer, the number of possible isomers increases exponentially with the chain length, which can be calculated: a given N-DP with x types of constitutive units and y types of linkages is able to generate N^x^ (N-1)^y^ possible isomers [48].

The presence or absence of modifications of the monomeric flavan-3-ol units further enhances the structure complexity of PAs. Various methyl, acyl or glycosyl substituents of the monomeric units of PAs all occur in natural products [28]. For example, the 3-hydroxyl group of the flavan-3-ol units of B-type PAs in grape seeds is usually esterified with gallic acid [1]. A recent report even shows evidence for the existence of galloylated A-type procyanidins in grape seeds [49]. Besides, free (-)-epigallo-catechin gallate is abundant in tea [50] and (-)-epicatechin gallate is contained in grape seeds [47]. Further more, gallic acid substituted (+)-catechin and (+)-gallocatechin also exist in grape [51,52]. In addition, glycosyl substituent is another common modification of the flavan-3-ol units, for the sugar is usually linked to the 3-hydroxyl group and also to the 5-hydroxyl group sometimes [53,54]. However, PA heterosides are less frequently detected than other flavonoid glycosides, such as anthocyanins.

It is noteworthy to point that as early as the 1970s, modern NMR technology was used to investigate the presence of PA dimers, and was used to demonstrate the phenomenon of their conformational isomerism and to propose two different forms of restricted rotation about IFL: one compact and the other extended [55]. In a rather recent study, through NMR and molecular modeling, researchers revealed the three-dimensional (3D) structures of five B-type PA dimers and their conformational exchange in water and hydro-alcoholic media. Interestingly, they found that the compact form for these PA dimers dominated in most cases and further discussed this finding in relation with tannin-saliva protein interactions which rendered PAs the flavor of astringency in wine [18].

It is well known that the structure determines the property for chemicals [56]. The structure complexity of PAs endows them with multiple biochemical properties, mainly including the interaction of proteins, the chelation of metals and the antioxidant bioactivity [57,58,59]. Surely, these essential properties are not only the basis of their various protective functions for plants, but also their potential nutritional effects for human beings. 

## General biosynthetic pathway of flavonoids

**Figure 3:**
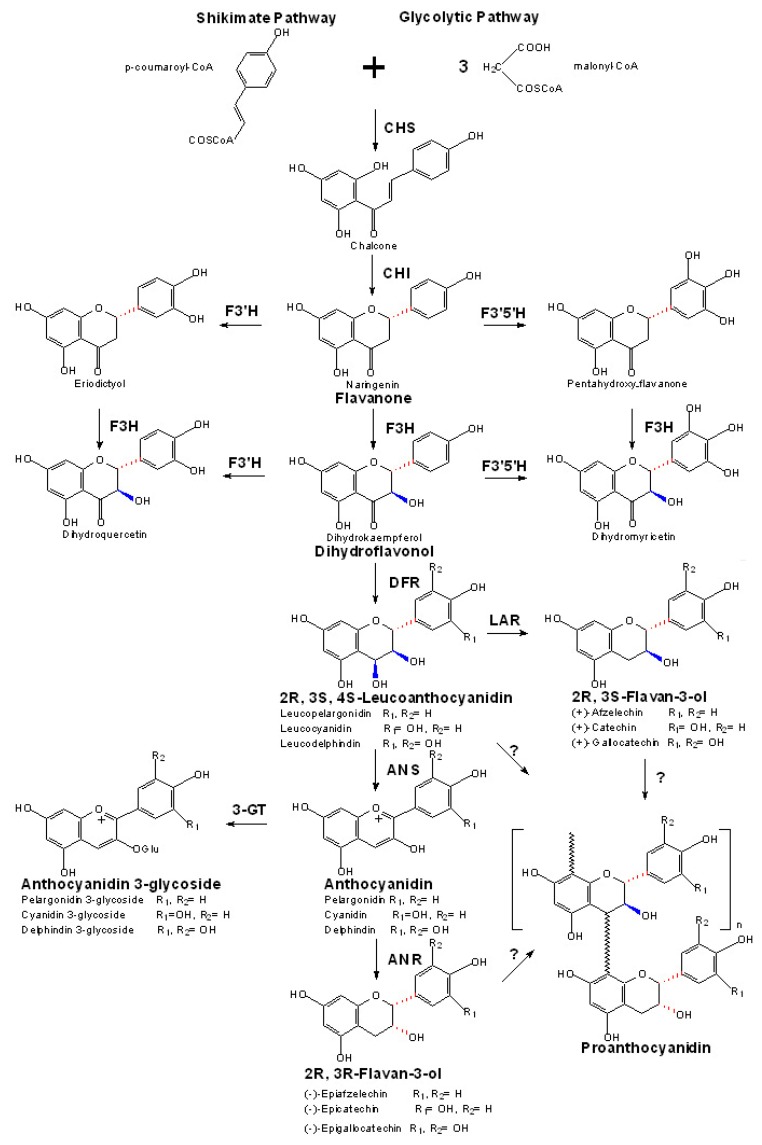
The general flavonoid pathway leading to the biosynthesis of PAs.

PAs are synthesized as oligomeric or polymeric end products of one of several branches of the flavonoid pathway, which shares the same upstream pathway with anthocyanins (Figure 3). Because of their great contribution to red color of plants, the biosynthesis of anthocyanins has been extensively investigated at the biochemical and genetic levels [60,61]. In recent years, the cloning and identification of a series of key structure genes relating to the formation of the direct precursors of PAs also lead us to a new era to understand the pathway [22]. Generally, investigations with plant mutants have largely promoted the identification of key structure genes in this pathway, as shown in Table 1 [62,63,64].

As is usual for flavonoid formation, the first committed step of the pathway is the condensation and subsequent intramolecular cyclization of three malonyl-CoA molecules with one 4-coumaroyl-CoA molecule to produce a naringenin chalcone [65]. This process is catalyzed by the ubiquitous plant enzyme chalcone synthase (CHS, EC 2.3.1.74, locus for *AtCHS*: At5g13930), which possesses extensive biological functions. CHS is found to mainly localize on the endoplasmic reticulum and tonoplast of the epidermal cells in *Arabidopsis* roots, and also in the chloroplasts or chromoplast of grape berry cells [66,67]. Additionally, the two kinds of precursors are derived from phenylalanine and acetyl-CoA, respectively.

The second step of the pathway is the isomerization of the naringenin chalcone to the naringenin, which can occur spontaneously, without enzymatic activity. However, chalcone isomerase (CHI, EC 5.5.1.6, locus for *AtCHI*: At3g55120) stereospecifically directs and greatly accelerates the intramolecular cyclization of chalcones to form the flavanones in the cytoplasm of plant cells (about 10^7^-fold more efficiently), which serve as exclusive substrates for downstream reactions [68]. In addition, the production of (2*S*)-flavanone by the catalysis of CHI is highly stereoselective, for the fact that pure CHI catalyzes the formation of (2*S*)-flavanone over 10^5^-fold faster than to the (2*R*)-flavanone and yields over 99.999% of the (2*S*)-flavanone in the products [69]. Following studies of the reaction mechanism of CHI also support the statement of that CHI controls the preferred formation of the biologically active (2*S*)-flavanones. For example, only the (2*S*)-isomer bounds in the CHI active site and the steric clash prevents the formation (2*R*)-naringenin [70,71]. Further more, the localization of CHI is also mainly on the endoplasmic reticulum and tonoplast of the epidermal cells in *Arabidopsis* roots, just like CHS [66]. Hither, the basic skeleton of all flavonoids which consist of three C6-C3-C6 aromatic rings has been generated through the catalysis of CHS and CHI.

**Table 1:** Partial mutants used for identifying structure genes in the general biosynthetic pathway of flavonoids in *Arabidopsis thaliana*, Maize and Barley.

Structure proteins	Plant species		
	Arabidopsis	Maize	Barley
CHS	*tt4*	*c2*	
CHI	*tt5*	*chi*	*ant30*
F3’H	*tt7*	*pr1*	*ant1, ant2, ant 5*
F3H	*tt6*	*fht1*	*ant17, ant22*
DFR	*tt3*	*a1*	*ant18*
ANS	*ans/tt18*	*a2*	
ANR	*ban*		
Transporter	*tt12*		

The following steps are related to the “Gird” section with the B-ring hydroxylation [22]. Flavonoid 3’-hydroxylase (F3’H, EC 1.14.13.21, locus for *AtF3’H*: At5g07990) or flavonoid 3’,5’-hydroxylase (F3’5’H, EC 1.14.13.88, no *F3’5’H* gene in *Arabidopsis thaliana*) can catalyze the conversion of naringenin into eriodictyol or pentahydroxyflavanone, respectively. These two enzymes both belong to the P450 monooxygenase superfamily of membrane-bound hemoproteins and work dependent upon NADPH and O_2_ [37,38]. All of the three (2*S*)-flavanones mentioned above can be oxidated to yield dihydroflavonols (dihydrokaempferol, dihydroquercetin and dihydromyricetin, respectively) stereospecifically by flavanone 3-β-hydroxylase (F3H, EC 1.14.11.9, locus for *AtF3H*: At3g51240), which is a non-heme iron enzyme and acts dependent upon Fe^2+^, O_2_, 2-oxoglutarate and ascorbate [72,73]. However, dihydrokaempferol can also be the potential substrate of F3’H or F3’5’H, which can convert it to dihydroquercetin or dihydromyricetin, respectively. Thus, both flavanones and dihydroflavonols can be hydroxylated by F3’H and F3’5’H, and the presence or absence of the two enzymes determines the B-ring hydroxylation pattern of dihydroflavonol, as well as the subsequent monomers in PAs and anthocyanins [37,38].

Following these, dihydroflavonol 4-reductase (DFR, EC 1.1.1.219, locus for *AtF3H*: At5g42800), depending upon NADPH, reduces dihydroflavonols (dihydrokaempferol, dihydroquercetin and dihydromyricetin) to form leucoanthocyanidins (also named flavan-3,4-diols, leucopelargonidin, leucocyanidin and leucodelphinidin, respectively) [74]. Different from the key enzymes in the early steps of the flavonoid pathway, DFR belongs to the plant reductase-epimerase-dehydrogenase (RED) superfamily. It is also worthwhile to point out that the conversion of (2*R*,3*R*)-dihydroflavonols to (2R,3S,4S)-leucoanthocyanidins by DFR is also stereospecific [75].

Anthocyanidin synthase (ANS, EC 1.14.11.19, Locus for *AtANS*: At4g22880), also known as leucoanthocyanidin dioxygenase (LDOX), is also a non-haem Fe^2+^, O_2_, 2-oxoglutarate and ascorbate dependent oxygenase [76]. ANS used to be recognized as the first pivotal enzyme of anthocyanin formation, but now it has been considered to play a significant role in PA biosynthesis as well [77]. In one pathway branch, leucoanthocyanidin molecules are oxidized by the catalysis of ANS to form colored anthocyanidins (pelargonidin, cyanidin and delphinidin, respectively) firstly [78]. These unstable anthocyanidins could be further converted into colorless (2*R*,3*R*)-flavan-3-ols [(-)-epiafzelechin, (-)-epicatechin and (-)-epigallocatechin, respectively] by the action of anthocyanidin reductase (ANR, EC 1.3.1.77, locus for *BAN*: At1g61720) [79,80]. Recently, researchers demonstrated that ANS from *Arabidopsis thaliana* is also able to convert its natural substrate leucoanthocyanidin to *cis*- and *trans*- dihydroquercetin *in vitro*, as well as quercetin, showing its flavonol synthase (FLS) activity with leucoanthocyanidin as substrate [81]. A more recent report reveals that beyond the oxidation of leucoanthocyanidins, ANS from *Gerbera hybrida* can also catalyze the oxidation of (+)-catechin to form an novel 4,4-dimer (93 %) and the conversion of (+)-catechin to cyanidin (7%) in *vitro*, suggesting its FLS activity with (+)-catechin as substrate [82]. 

In another pathway branch, leucoanthocyanidins can be converted into (2*R*,3*S*)-flavan-3-ols [(+)-afzelechin, (+)-catechin and (+)-gallocatechin, respectively] by leucoanthocyanidin reductase (LAR, EC.1.17.1.3, no *LAR* gene in *Arabidopsis thaliana*) [83]. Interestingly, some plants such as *Vitis vinifera*, *Gossypium arboretum* and *Gossypium raimondii*, contain two homologous *LAR* genes, whereas other plants such as *Hordeum vulgare*, *Phaseolus coccineus*, *Pinus taeda*, *Vitis shuttleworthii* only have a single *LAR* gene in the cells [84]. No *LAR* gene has ever been found in *Arabidopsis thaliana,* so PAs in this plant are composed of only (-)-epicatechin units [1]. Both LAR and ANR are two members of the isoflavone reductase-like (IFR-like) group of the plant RED super family, which are both localized in the cytosol [84,85]. Thus, the two familiar potential precursors, 2,3-*cis*-2*R*,3*R*-(-)-epicatechin and 2,3-*trans*-2*R*,3*S*-(+)-catechin are synthesized from two distinct pathway branches by two different substrates and two distinct enzymes, although the difference between these two potential precursors is only in the *cis*- or *trans*- stereochemistry at the C2 and the C3 positions of the C-ring [1]. However, there are still no exact answers at present to the existence and the formation of the biosynthetic *ent*-flavan-3-ols, including 2,3-*cis*-2*S*,3*S*-(+)-epicatechin and 2,3-*trans*-2*S*,3*R*-(-)-catechin. 

Additionally, the unstable anthocyanidins can be also glycosylated at the 3-*O*-position to produce their corresponding anthocyanidin 3-glycosides by the catalysis of UDP-glucose:anthocyanidin/ flavonoid 3-glucosyltransferase (UFGT or 3-GT, EC 2.4.1.115, locus for *AtUFGT*: At5g54060), leading to the absolute branch pathway of anthocyanin biosynthesis [86,87].

Both (2*R*,3*R*)-flavan-3-ols and (2*R*,3*S*)-flavan-3-ols, as well as (2*R*,3*S*,4*S*)-flavan-3,4-diols are proposed as the potential precursors of PAs. However, the identity of the final “enzyme” which catalyzes the polymerization reaction of these precursors to form PAs and the exact mechanism whereby the elementary units of flavanols are assembled *in vivo* remain unknown [1,22,23]. Thus, a unilateral genetic or molecular genetic research approach seems to be not enough, and the debate concerning the operation of an enzymatic or nonenzymatic mechanism for PA condensation still continues. Recently, a plasma membrane H^+^-ATPase (AHA10, locus for *AtAHA10*: At1g17260) is found to be required for the formation of PAs in the seed coat endothelium of *Arabidopsis thaliana*, the mutant of which can disrupt both the PA biosynthesis and the vacuole biogenesis. Further studies support the hypothesis that AHA10 or even other P-type proton pumps in plants could help acidify cytoplasmic or vacuolar compartments [88]. According to the proposed mechanism of acid catalysis of PA formation, flavan-3-ol (terminal unit) can be added to a quinone methide or its protonated carbocation (the precursor of extension unit) nonenzymatically under acidic environment [22]. Thus, the founding of AHA10 is essential to promote such nonenzymatic polymerization mechanisms for PAs.

## Transport factors of proanthocyanidin precursors

Generally, the known enzymes in the biosynthetic pathway of flavonoid are observed to mostly locate on the endoplasmic reticulum membranes or in the cytoplasm [66]. However, it is well known that PAs accumulate in the vacuole. Thus, the intracellular transport of PA precursors, flavan-3-ols and/or flavan-3,4-diols from the site of synthesis to the site of storage is a crucial problem in PA biosynthesis.

Up to now, only two genes involved in transport processes required for PA biosynthesis have been identified from the seed coat of *Arabidopsis thaliana* by genetic analysis methods. Both of the genes are obtained from the *transparent testa* mutations (light-colored seeds) which are caused by reductions of PA deposition in vacuoles of endothelial cells, and are named as *tt12* and *tt19*, respectively. However, the functional relation between TT12 and TT19 remains unclear until now [89,90].

The *TT12* gene (locus: At3g59030) encodes a protein with 12 transmembrane segments exhibiting similarity to prokaryotic and eukaryotic secondary MATE (multidrug and toxic compound extrusion) transporters. This gene is expressed specifically in the endothelial layer of the developing seed coats, suggesting that TT12 may be involved in the accumulation of PA precursors in vacuoles [89]. Further studies reveal that flavan-3-ols and PAs are absent, and quercetin-3-*O*-rhamnosides are reduced in the vacuoles of *tt12* mutant seeds, and this transporter is localized to the tonoplast. Finally, TT12 is demonstrated to act as a vacuolar flavonoid/H^+^-antiporter on the vacuolar membrane of PA-synthesizing cells of the seed coat. It mediates the *MgATP*-dependent transport of cyanidin-3-*O*-glucoside in *vitro* but not the aglycones cyanidin and epicatechin. Furthermore, TT12 transports neither glycosylated flavonols and procyanidin dimers nor nonglycosylated flavan-3-ol monomers and dimers [91]. As a consequence, a series of new questions about the biosynthesis of PAs are put forward.

The *TT19* (locus: At5g17220) gene is a member of the *Arabidopsis thaliana* glutathione *S*-transferase (GST) gene family, which is required for transferring anthocyanins into vacuoles. Because the deposition pattern of PA precursors in the *tt19* mutant is also different from that in the wild type, TT19 may participate in the PA pathway with an unclear role in flavonoid accumulation [90]. Similar GST genes are also found in other plants. In maize, the *Bronze-2* gene encoding a GST is related to the deposition of red and purple pigments (anthocyanins) in the vacuoles of maize issues [92]. In petunia, the *AN9* gene encoding a GST involved in anthocyanin transport is quite similar to the *TT19* gene functionally, which can complement the *Arabidopsis thaliana tt19* mutation with respect to allowing vacuolar uptake of anthocyanins, but can not restore the deposition of PAs [93]. Recently, four specific genes are identified as systematically co-expressed with anthocyanin accumulation in grape berries. One of them is a type-I GST gene which is orthologous to the *Bronze-2* gene and the *AN9* gene and encodes a flavonoid binding GST protein required for vacuolar transport of anthocyanins [94].

However, no such MATE transporter factors have been found in grapes until now. Interestingly, some very recent research shows the evidence for a putative flavonoid translocator similar to mammalian bilitranslocase (BTL, TC 2.A.65.1.1) in grape (*Vitis vinifera* L.) berries of cultivar Merlot (red grapes) during ripening, which is responsible for the transport of anthocyanins [95].

## Potential polymerization mechanisms for proanthocyanidins

Although the biosynthesis of the precursors of PAs has been well known for several years, a clear polymerization mechanism is still unknown [23]. Most of models state that the electrophilic C4 position of the extension unit (flavan-3,4-diol) condenses with the nucleophilic C8 or C6 position of the start/terminal unit (flavan-3-ol) to produce PAs [96,97]. However, these models neglect the fact that the enzymatic formation of flavan-3,4-diol is stereospecific 2,3-*trans*, whereas lots of the extension units are 2,3-*cis* in plants [75]. To support this, Porter characterized 58 procyanidin oligomers from plants of more than two dozen different species, and found that (-)-epicatechin accounted for 55% and 81% as the start and extension units, respectively [98,99]. This paradox has been standing for a long time and partially been solved by the functional identification of *BANYULS* in recent years [79]. Up to now, some highly speculative solutions have been developed for the mechanism of PAs condensation.

**Figure 4:**
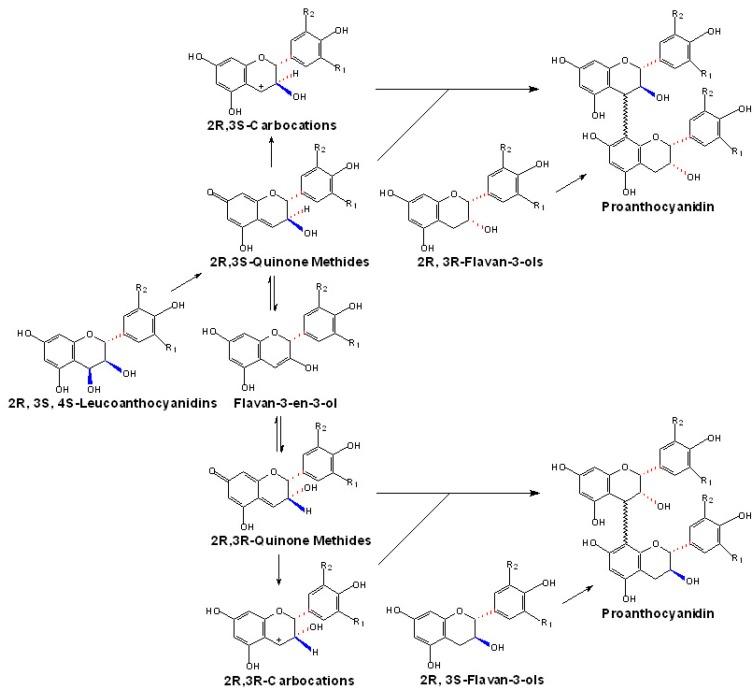
Putative route of the generation of quinone methides or carbocations from flavan-3,4-diols.

In one previously proposed route, 2*R*,3*S*,4*S*-leucoanthocyanidins are first converted into 2R,3S-quinone methides, which can be used as the 2*R*,3*S*- extension units directly. Meanwhile, they can also be converted into 2*R*,3*R*-quinone methides via the flavan-3-en-3-ol intermediates, which can be condensed as the 2*R*,3*R*- extension units directly [100]. Furthermore, these *trans-* or *cis-* quinone methides could be converted to their corresponding carbocations which could be attacked by (+)-catechins or (-)-epicatechins directly to produce the PAs (Figure 4). However, these hypotheses are lack of direct experimental proof in *vivo* until now, and the only experimental basis comes from the reports of Creasey and Swain, who first chemically synthesized leucocyanidin from (+)-dihydroquercetin and make it possible to synthesize procyanidins through the condensation reactions of leucocyanidin with either (+)-catechin or (-)-epicatechin *in vitro* [101].

**Figure 5:**
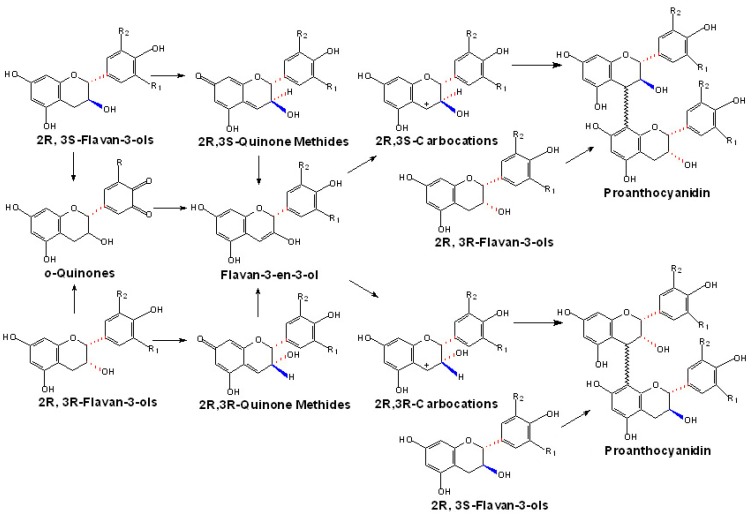
Putative route of the generation of quinone methides or carbocations from flavan-3-ols.

In another model, flavan-3-ols [(+)-catechins or (-)-epicatechins] are converted to their corresponding quinone methides by the catalysis of polyphenol oxidase (PPO). These quinone methides can be further converted to carbocations via flavan-3-en-3-ol intermediates or be reduced to carbocations through coupled non-enzymatic oxidation, and these carbocations can be accepted as the direct extension units [1]. Interestingly, *o*-quinones are also supposed to be derived from (+)-catechins or (-)-epicatechins by enzymatic or nonenzymatic reduction [1]. And also via flavan-3-en-3-ol intermediates, *o*-quinones could be converted to their corresponding carbocations which may participate in the condensation reaction (Figure 5). However, this hypothesis is highly speculative and does not have any direct experimental support.

The general localization of PPO is on the plastids (or chloroplasts), whereas the formation and accumulation of PAs is in the vacuoles [102]. Therefore, the model of PPO mediated PA polymerization requires the existence a novel form of PPO with alternative localization [1]. Recently, aureusidin synthase, a flavonoid biosynthetic PPO that catalyzes the oxidative formation of aurones from chalcones in snapdragon (*Antirrhinum majus*) and is responsible for the yellow coloration of flowers, is found to localize within the vacuole lumen, showing us the variability of the PPO localization [103]. 

**Figure 6:**
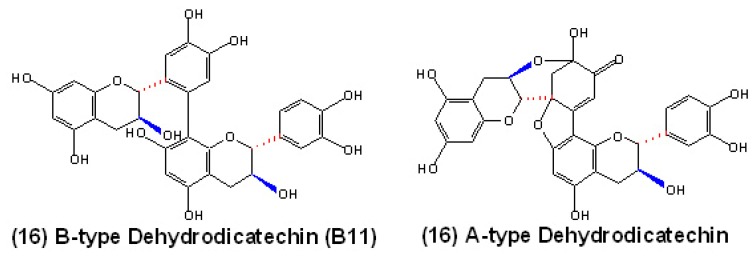
Structures of PPO mediated oligomeric flavan-3-ols: B-type (16) and A-type (17) dehydrodicatechins.

However, in the PPO mediated oxidative polymerization of flavan-3-ols, researchers do not obtain PAs with natural structures, but rather a series of oxidative flavan-3-ols oligomers with particular configurations. For example, in the oxidation of (+)-catechins in aqueous systems carried out using grape PPO as catalysts, some yellow pigments and colorless products of oligomeric (+)-catechins are formed [104]. In another report of Oszmianski and Lee, the enzymatic oxidation of (+)-catechin, chlorogenic acid and their mixture with PPO also produce unnatural dimers and other polymers of (+)-catechin, polymers of chlorogenic acid and copolymers of (+)-catechin and chlorogenic acid, respectively [105]. Here, such oxidative dimers of flavan-3-ols are identified as dehydrodicatechins, which are usually formed as a result of enzymatic oxidation, chemical oxidation or autoxidation of flavan-3-ols [104,106,107]. Generally, the oxidative dimers of flavan-3-ols linked by C6’→C8 or C6’→C6 IFL are classified as B-type dehydrodicatechins, resulting from the repeated condensation reactions between the A-ring of the lower unit and the B-ring of the upper unit through a mechanism of so-called ‘‘head to tail’’ polymerization. Correspondingly, the oxidative dimers which contain additional C-O-C ether-type IFL are classified as A-type dehydrodicatechins, as shown in Figure 6 [104]. Furthermore, a new *TT10* gene is obtained through the *Arabidopsis thaliana tt10* mutant, which encodes a protein that may be involved in the oxidative polymerization of flavonoids and functions as a laccase-type flavonoid oxidase. Interestingly, the major products resulting from TT10 activity are also yellow quinone-methide epicatechin dimers and trimers, which are not characterized but possibly related to dehydrodicatechins [108]. Therefore, the model of PPO mediated PA polymerization can not obtain direct experimental support from the previous studies, and PAs might not be simply formed through the PPO-mediated condensation mechanism.

Anthocyanins and anthocyanidins are other groups of potential substrates for PA polymerization [1]. On one hand, anthocyanins are shown to condense with PA monomers (flavan-3-ols), oligomers or polymers in wine to change its tannin composition, as well as its color and mouth-feel [109,110]. And on the other hand, anthocyanidins are mainly present as their corresponding flavylium ions under acidic conditions. These ions may be converted to quinone methides and then to carbocations depending upon PPO [111]. Carbocations can condense with each other as the extension units, and also condense with flavan-3-ols through non-enzymatic oxidation [112].

Furthermore, the biogenesis of 2,3-*cis*-2*R*,3*R*-PAs from 2,3-*trans*-2*R*,3*R*-dihydroflavonols may also be accounted for by tautomerism between quinone methide and flav-3-en-3-ol intermediates [113]. Although flavan-3-en-3-ols may be released from ANR theoretically, no dimeric or oligomeric PAs are detected to form with ANR reactions in *vitro* [79].

## Regulation of proanthocyanidins biosynthesis

Similar to other secondary metabolism pathways in plants, the biosynthetic pathway of PA is under complex control by multiple regulatory genes at the transcriptional level [27]. According to gene structure, these regulatory genes are categorized into six different families: Myc transcriptional factors (encoding basic helix–loop-helix proteins, bHLH), Myb transcriptional factors, WD40-like protein, WRKY transcription factors, MADS homeodomain genes and TFIIIA-like proteins “WIP”, as summarized in Table 2 [22]. 

Generally, *Myc* genes, together with other regulatory genes such as *Myb* genes, make a widely control of the biosynthesis of PAs, as well as some other flavonoid end products like anthocyanins [114,115,116,117]. WD40-like protein is required for the accumulation of anthocyanins and PAs, as well as the formation of root hair and trichome [118]. Recently, researchers found that in *Arabidopsis thaliana*, several Myb and Myc proteins form ternary MYB-BHLH-WDR (MBW) complexes with WD-repeat proteins that regulate the transcription of genes involved in biosynthesis of anthocyanins and PAs [119]. For example, TT2 (Myb family), TT8 (Myc family), and TTG1 (WD-like protein) form a transcriptional complex capable of directly activating the expression of *BAN* [120]. WRKY transcription factor is a zinc finger-like protein which acts downstream of WD40-like protein, and also affects the accumulation of PAs [121]. Furthermore, a recent study also suggests that the WRKY transcription factor is directly regulated by Myb transcription factors, such as TT2, etc [122]. MADS homeodomain genes directly regulate *BAN* and may act upstream of other regulatory genes, but they affect the accumulation of PAs regionally [123,124]. WIP proteins specifically control the assembly of PAs polymer, rather than the formation of monomeric flavan-3-ol units [125]. Additionally, WIP proteins also interact with developmental genes, such as *BAN,* to control PA biosynthesis. However, besides these six major families, there are also regulatory genes of other types which also have direct or indirect influences on the genes of biosynthesis of anthocyanins and/or PAs, such as *ANTHOCYANINLESS2* (*ANL2*) which is a homeobox gene of the homedomain-leucine zipper (HD-ZIP IV) family, AtDOF4;2 which is a member of DNA-binding-with-one-finger (DOF) transcription factor family, etc [126,127].

**Table 2:** Transcription factors involved in proanthocyanidin biosynthesis in *Arabidopsis thaliana*.

Families	Genes	Locus	Gene regulated
MYC	*TT8*	At4g09820	*BAN*, *DFR*
	*GL3*/*EGL3*	At5g41315/At1g63650	*DFR*
MYB	*TT2*	At5g35550	*BAN*, *DFR*, *ANS*, *TT12*, *AHA10*, *TT8*, *TTG2*
	*PAP1*/*PAP2*	At1g56650/At1g66390	*PAL*,*CHS*, *DFR*, *ANS*, *TT8*
WD40	*TTG1*	At5g24520	*BAN*, *DFR*, *TT8*, *TTG2*
WRKY	*TTG2*	At2g37260	*BAN*, *TT12*
MADS	*TT16*	At5g23260	*BAN*, *TT2*
WIP	*TT1*	At1g34790	*BAN*

On the other hand, these regulatory genes can also be classified into three different categories according to their functions: (1) regulation of the general genes in the pathway of flavonoid biosynthesis down to anthocyanidins, (2) modulation of the branch pathway of PAs, including the biosynthesis, transport and polymerization of the subunits of PAs; and (3) control of the developmental processes for the generation of the cells accumulating PAs specially [1]. So far, most of the regulatory genes and regulation mechanism have been characterized in *Arabidopsis thaliana* by using several mutants with low level of PAs or with low levels of both anthocyanins and PAs. In *Arabidopsis thaliana*, the first category of transcriptional factors which regulate the general flavonoid biosynthesis down to anthocyanidins includes *PAP1 & PAP2* (Myb family), *GL3 & EGL3* (Myc family) and *TTG1*, which are connected in a hierarchical network to regulate flavonoid synthesis [115,118,119,128]. The second category of regulatory genes in *Arabidopsis thaliana* includes *TT2*, *TT8* and *TTG1*, modulating the expression of the late flavonoid biosynthetic genes, such as *DFR*, *ANS*, *BAN*, the transport factor *TT12* and the proton pump *AHA10* [116,118,129]. The third category of transcription factors includes *TT1*, *TT16* and *TTG2 in Arabidopsis thaliana*, and they regulate organ and cell development for PA deposition, as well as the transcription of PA specific genes [121,122,123,125]. 

It is worth pointing out that a series of *Myb* and *Myc* genes are found to work as negative regulators of the biosynthetic pathway of anthocyanins and PAs in *Arabidopsis thaliana*. *MYBL2* encodes a R3-MYB-related protein that interacts with MBW complexes and directly modulates the expression of flavonoid target genes. The loss of MYBL2 activity leads to a dramatic increase in the accumulation of anthocyanins, while overexpression of MYBL2 in seeds inhibits the biosynthesis of PAs, showing its negative regulation on the pathway [130]. MYB4 is a R2R3-MYB that can interact with BHLH proteins controlling the expression of flavonoid structural genes and repress the transcription of early genes in phenylpropanoid metabolism [131,132]. Myc negative regulators affecting flavonoid TTG1-dependent pathways also exist in *Arabidopsis thaliana*. *BHLH32* in *Arabidopsis* negatively regulates root hair formation, anthocyanin accumulation and phosphoenolpyruvate carboxylase kinase (*PPCK*) expression, all of which are induced by (inorganic phosphate) Pi starvation. For example, *BHLH32* seems to act as a negative regulator of *DFR* in Pi-sufficient conditions [133].

**Figure 7:**
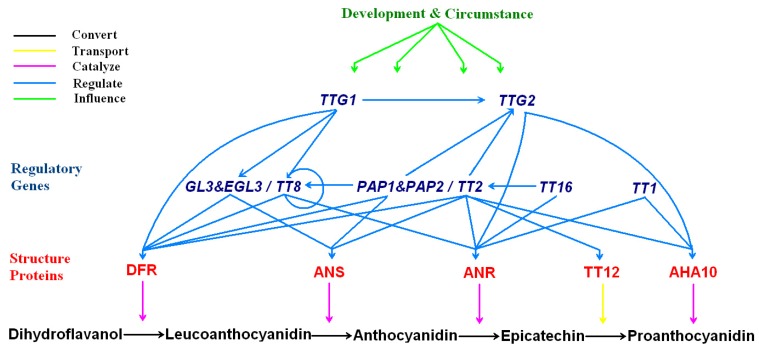
Interactions among the transcription factors and the structure proteins in *Arabidopsis thaliana.*

The relationship between the transcription factors and the structure proteins in *Arabidopsis thaliana* is illustrated in Figure 7. As shown, a complex regulatory network controls PA accumulation in plants [134]. Almost all of these transcription factors regulate the expression of *BAN*, suggesting its essential function among the structure genes in the biosynthetic pathway. Meanwhile, *TT2*, *TT8* and *TTG1* play a central role in the regulation of the PA biosynthesis and have complicated interactions. For example, *TT8* controls its own expression in a feedback regulation involving *TTG1* and homologous MYB (*TT2*, *PAP1*) and MYC (*TT8*, *GL3* and *EGL3*) factors [128,129]. However, the direct or indirect regulation of these regulators by internal or external signals leading to controlled responses is more perplexing [135]. In plants, it is well known that fertilization is a key signal to start the PA biosynthesis, as it is required for the differentiation of PA-accumulating cells and the activation of the promoter of *BAN*, as well as most of other specific structure genes [84,136,137]. Additionally, more and more environmental factors are found to have significant influences on the biosynthesis of PA and anthocyanins in plants, such as light stress and shading, atmospheric change (CO_2_, N_2_, O_2_ and O_3_), temperature (day and night), exogenous plant hormones (abscisic acid, naphthaleneacetic acid and ethylene), infection of pathogens (bacteria and fungal), UV (UV-A and UV-B) or solar radiation, nitrogen, water and Pi deficiency, etc. These environmental factors do not only manipulate the expression of the structure genes, but also widely affect the functions of the transcription factors [138,139,140,141,142,143,144,145,146,147,148,149]. 

Similar regulatory genes are also identified in maize, which are coordinately active at the transcriptional level especially in the aleurone and belong to the first three types: Myc transcriptional factors (*R*, *B-Peru*, *Sn*, *Lc*), Myb transcriptional factors (*Cl*, *P*) and WD40-like protein (*PAC1*) [150,151,152].

However, until now, only one major class of regulatory genes related to PA biosynthesis has been found in grape: the Myb transcriptional factors. VvMYBPA1, which was cloned and characterized from grapevine (*Vitis vinifera* L.) tissues of cultivar Shiraz (red grapes), controls the expression of the pathway genes of PAs including both *VvLAR1* and *VvANR,* but do not activate the promoter of *VvUFGT* which is considered as an anthocyanin specific gene. That means VvMYBPA1 is specific to regulate the biosynthesis of PAs in grape [153]. Another group of Myb family transcriptional factors in grape named as VvMYBA, including VvMYBA1, VvMYBA2 and VvMYBA3, are only expressed after véraison (grape color change) and demonstrated to regulate anthocyanin biosynthesis during ripening [154,155,156]. VvMYB5a is the third grape transcriptional factor that is expressed mainly at the early steps of berry development (prior to véraison) and affects the metabolism of a series of polyphenols, including flavanols, anthocyanins, PAs and even lignins. It is thus suggested that VvMYB5a regulates a series of branches of phenylpropanoid pathway in grapevine, but not only that of flavonoids [157].

## Genetic manipulation of proanthocyanidin biosynthesis

PAs have been recognized to play a significant role in the prevention of pasture bloat and the enhancement of bypass proteins [158,159]. Thus, the induction of PA biosynthesis in some forages, such as alfalfa (*Medicago sativa* L.), can largely solve the problems of pasture blot and protein loss [158]. On the other hand, PAs also have negative influences on non-ruminant livestock, because of their properties of non-specific interaction with protein and complexation of metal ions [160,161]. As a result, the reduction of PA content in some crops, such as canola (*Brassica napus* L.), can also increase their values for non-ruminant livestock feed. However, it is difficult to manipulate the content of PAs in plants by using conventional breeding strategies, such as induced mutation, somaclonal variation and somatic hybridization [20]. Genetic manipulation of PA biosynthesis has its unique advantage, and a lot of the efforts have been drawn to manipulate the PA biosynthesis in the forage legumes, such as alfalfa and *Lotus corniculatus* (bird’s foot trefoil). Generally, the genes of both structure enzymes and transcription factors are used in the attempts to genetically engineer PA biosynthesis [162,163,164,165,166,167,168]. 

Three maize anthocyanin regulatory genes *C1* (Myb family), *Lc* and *B-peru* (Myc family), are all introduced respectively to the genetic alternation of alfalfa which does not produce PAs naturally. However, only in the *Lc*- transgenic alfalfa both anthocyanins and PAs are accumulated in leaf tissue under stress conditions of high light intensity or low temperature [162]. These results indicate that PA biosynthesis can be stimulated by some Myc transcription factors in alfalfa, and offer us a feasible way to obtain bloat-safe alfalfa forage for ruminants [163].

In contrast, much more efforts have been put into the genetic engineering of that in *Lotus corniculatus*, a common forage legume which naturally produces PAs. Antisense down-regulation of *DFR* expression successfully reduces its PA content of up to ≈80% in hair root cultures, but over-expression of *DFR* in hairy roots results in variable influences on PA content and its monomer composition [164,165]. In another research, over-expression of a maize anthocyanin regulatory gene *Sn* (Myc family) leads to unexpectedly increase in PA accumulation and to dramatically increase in the number of tannin-containing cells in its tissue [166]. Further studies suggest that light and the transgene *Sn* synergistically affects the PA biosynthesis, as well as the structure genes in the pathway, such as *DFR*, *ANS*, *ANR* and *LAR* [167,168].

Beside these, a lot of attempts have been performed in a series of model plants, such as *Arabidopsis thaliana*, tobacco and *Medicago truncatula*, the purpose of which is mainly to clarify the exact functions of the structure genes and the transcription factors, or their functional relations [169,170]. In addition, it is worthwhile to mention that although grapevine is not suitable for being manipulated as the target plant, it is an ideal choice as the gene source, especially for the regulatory genes. Actually, some of Myb transcription factors in grapevine have already been introduced to other model plants. For example, the ectopic expression of *VvMYB5a* in tobacco has been used in the functional identification of this gene, and the *VvMYBPA1* gene can complement the Arabidopsis *TT2* gene in its PA deficient mutant [153,157].

## Discussion and Conclusions

In the past few years, our knowledge of the mechanism of PA biosynthesis has been changed by a series of advances. Nowadays, researchers have a deep understanding of the structure diversity of PAs, find and identify a host of structure genes and their regulatory factors, and even genetically manipulate the accumulation of PAs or other end products of flavonoid pathway in some plants. However, a number of important questions remain unclear, some of which have been discussed emphatically by previous researcher, as illustrated below [1,22,23]. 

What mechanism controls the flux at the interface between the anthocyanin and PA pathways, the known structure proteins and/or the regulatory factors? What are the functional relationships between them? Do the circumstance factors have influence on the flux control?

Is LAR unnecessary for the biosynthesis of PAs, as its absence in *Arabidopsis thaliana*, as well as its product of (+)-catechin? Is this phenomenon a sign of evaluation for plants, or not? How are LAR and ANR regulated at the protein level (translation level)?

What are the exact extension units of PAs, flavan-3-ols, flavan-3,4-diols or their derivates? Does the monomer availability mainly restrict the diversity of the PA composition? How are the *ent*-flavan-3-ols formed, by nonenzymatic isomerization or enzymatic alternation?

How exactly are the PA precursors transported into the vacuole? Is TT12 the only MATE transporter in plants? Does it cooperate with TT19? How are these glycosylated cyanidin monomers used in vacuole? Is there any homologous transporter which allow vacuolar uptake of nonglycosylated flavan-3-ol monomers as the direct precursors for PAs? 

How PA oligomers or polymers are actually assembled *in vivo*? Is it a nonenzymatic process or operated by one or more structure proteins? If it belongs to an enzymatic mechanism, what are the exact compositions of these enzymes? Is it a polymerase for the direct condensation, or a PPO for the formation of quinone methides? Furthermore, what is the location for the condensation within the vacuole?

We speculate that the answers to the above questions will be obtained depending upon the applications of the biochemical, genetic and biogenetic technologies in no distant further, for example, the screening of the mutants specific to PA condensation in *Arabidopsis thaliana* or barley, the preparation and application of the antibodies of the key enzymes or transcription factors involved in the biosynthesis of PAs, the application of technologies of protein-protein interaction, etc [171,172,173]. On the other hand, further achievements in other areas of PA studies will also facilitate and accelerate our understanding of this group of polyphenolic secondary metabolites to extent our eyeshot of the natural products and to improve our daily life.
